# Importance of *PNO1* for growth and survival of urinary bladder carcinoma: Role in core‐regulatory circuitry

**DOI:** 10.1111/jcmm.14835

**Published:** 2019-12-04

**Authors:** Chunhua Lin, Hejia Yuan, Wenting Wang, Zhe Zhu, Youyi Lu, Jiahui Wang, Fan Feng, Jitao Wu

**Affiliations:** ^1^ Department of Urology The Affiliated Yantai Yuhuangding Hospital of Qingdao University Yantai China; ^2^ The Central Laboratory The Affiliated Yantai Yuhuangding Hospital of Qingdao University Yantai China; ^3^ Division of Regenerative Medicine Department of Medicine School of Medicine University of California, San Diego La Jolla CA USA

**Keywords:** bladder cancer, DNA microarray, ingenuity pathway analysis, *PNO1*

## Abstract

*PNO1* (partner of Nob1) was known as a RNA‐binding protein in humans, and its ortholog *PNO1* was reported to participate ribosome and proteasome biogenesis in yeasts. Yet there have been few studies about its functions in mammalian cells, and so far its role in human cells has never been reported, especially in urinary bladder cancer (UBC).We interrogated the cellular functions and clinical significance of PNO1 in, and its molecular mechanism through microarrays and bioinformatics analysis. Our findings support that *PNO1* participates in promoting proliferation and colonogenesis, while reducing apoptosis of UBC cells, and is also predicted to be associated with the migration and metastasis of UBC *PNO1* knockdown (KD) attenuated the tumorigenesis ability of UBC in mouse. *PNO1* KD led to the altered expression of 1543 genes that are involved in a number of signalling pathways, biological functions and regulation networks. CD44, PTGS2, cyclin D1, CDK1, IL‐8, FRA1, as well as mTOR, p70 S6 kinase, p38 and Caspase‐3 proteins were all down‐regulated in *PNO1* KD cells, suggesting the involvement of *PNO1* in inflammatory responses, cell cycle regulation, chemotaxis, cell growth and proliferation, apoptosis, cell migration and invasiveness. This study will enhance our understanding of the molecular mechanism of UBC and may eventually provide novel targets for individualized cancer therapy.

## 1 INTRODUCTION

Urinary bladder cancer (UBC) is the ninth most common malignancy and the thirteenth most common cause of cancer‐related death worldwide.^1^ In 2018, there have been an estimated 81 190 new cases of UBC in the United States, causing a total of 17 240 deaths, with the incidence ratio of male vs female being 3.3 vs 1.^2^ In China, the total incidence of UBC was 80 500, causing 32 900 deaths annually, with the male vs female ratio being 3.4 vs 1.^3^ UBC is a highly immunogenic cancer with a higher rate of mutation than other types of cancers.^4^ The current trend of ‘personalized and precision care’ in cancer therapy requires multiple layers of molecular profiling of biomarkers for accurate diagnosis and prediction of treatment responses.^4^ It is therefore important to identify novel biomarkers and oncogenes for UBC and thus can improve personalized and precision care.

*PNO1* was first characterized as a novel RNA‐binding gene partner of *NOB1* isolated from human kidney.^5^ *PNO1* is highly conserved, all the way from yeasts up to mammals.^6^ In yeasts, *PNO1* is reportedly involved in both ribosome and proteasome biogenesis.^7^, ^8^ In mammalian cells, it is localized to the nucleus, especially within the nucleoli.^5^ In humans, *PNO1* is most abundantly expressed in the thyroid, adrenals, appendix, placenta, bone marrow, urinary bladder and testes (NCBI Gene Database, ID: 56902). Currently, there have been few studies about its functions in mammalian cells, and so far its role in humans has not been reported.

To this end, the aim of the study was to identify the potential involvement of *PNO1* in human UBC. The association of *PNO1* with UBC was studied both in vitro and in vivo, and its molecular mechanism was predicted through microarray and bioinformatics analysis.

## 2 MATERIALS AND METHODS

The human and animal subjects and materials of the paper were approved by the Yantai Yu Huang Ding Hospital's ethical committee.

### 2.1 Cell culture knockdown of PNO1 by lentivirus

T24 and 5637 bladder cancer cells were routinely cultured in an RPMI‐1640 medium (Gibco), supplemented with 10% foetal bovine serum (Gibco) at 37°C in 5% CO_2_ humidified incubator. Cells were harvested in a logarithmic phase of growth for all experiments.

Lentivirus carrying the *PNO1* gene interfering shRNA sequence (shPNO1, target sequence 5′‐TGAACAATTTCAGTCATTT‐3′) or non‐silencing control (shCtrl, target sequence 5′‐TTCTCCGAACGTGTCACGT‐3′) was constructed by GeneChem, Shanghai, China. Cells were seeded in plates and grown to a density of 15%‐30% in good conditions, before being infected with the above‐mentioned lentivirus (containing fluorescence), according to the manufacturer's protocol. The culture medium was changed to normal medium 8‐12 hours after infection. Cells were observed 72 hours post‐infection with fluorescent microscope to ensure a positive infection rate of >70%.

### 2.2 RNA isolation and quantitative real‐time PCR (qRT‐PCR)

Total RNA was extracted from cells using SuperfecTRI total RNA isolation reagent (Pufei), according to the manufacturer's instructions. The concentration of RNA was determined by spectrophotometry (Nanodrop 2000/2000C, Thermo Scientific). The total RNA was then reverse‐transcribed using M‐MLV Reverse Transcriptase (Promega). qRT‐PCR analysis was performed on a LightCycler® 480 System (Roche) with SYBR Master Mixture (DRR041B, TAKARA) according to the manufacturer's protocol. Cycling conditions were as follows: 95°C for 30 seconds, followed by 40 cycles of 95°C for 5 seconds, and then 60°C for 30 seconds. *GAPDH* was used as endogenous reference. ΔCt (CtPNO1 − CtGAPDH) ≤ 12 suggested high abundance expression. −ΔΔCt = average ΔCtshCtrl − ΔCtshPNO1. 2−ΔΔCt represented the relative expression of *PNO1* in *PNO1* knockdown cells compared with control cells.

### 2.3 Western blot

Cellular protein extraction and Western blot were performed as previously reported.^9^ Proteins were identified with antibodies from Santa Cruz Biotechnology: rabbit anti‐PNO1 (sc‐133263), mouse anti‐GAPDH (sc‐32233), goat anti‐rabbit IgG‐HRP (sc‐2004) and goat antimouse IgG‐HRP (sc‐2005); from Abcam: rabbit anti‐CD44 (ab 51037), rabbit recombinant Tissue Factor antibody (F3, ab151748), rabbit anti‐CDK1 (ab32094), rabbit anti‐FRA1 (FOSL1, ab124722), rabbit anti‐COX2 (ab15191) and mouse anti‐IL8 (CXCL8, ab18672); or from Cell Signaling Technology: rabbit anti‐CCND1 (#2978).

### 2.4 Automated cell counting

Lentivirus‐infected cells were seeded with GFP fluorescence in plates at an appropriate concentration and cultured under routine conditions. Plates were read on a *Celigo*® Image Cytometer (Nexcelom Bioscience) every day for 5 days, according to the manufacturer's instructions, to obtain the number of fluorescent cells at each time‐point. Growth curves based on cell counts or fold changes against the number of cells on day 1 were plotted.

### 2.5 MTT assay

Cell viability was analysed using an MTT assay. Two thousand cells were seeded in each well of the 96‐well plates and cultured under routine conditions. At each time‐point, 20 μL of 5 mg/mL MTT (Genview) was added to each well, followed by a 4‐hour incubation after which the culture medium was replaced by 100 μL DMSO. Following 2‐5 minutes agitation, the optical densities at 490 nm wavelength (OD490) were measured on an Infinite® M2009PR microplate reader (Tecan). Growth curves based on OD490 or fold changes against the OD490 on day 1 were plotted.

### 2.6 Colony formation assay

Cells infected with lentivirus for 3 days were seeded in 6‐well plates at a concentration of 800 per well and cultured for around 10 days. Cells in each well were fixed with 1 mL 4% paraformaldehyde for 30‐60 minutes and stained with 500 μL GIEMSA Stain (Sigma‐Aldrich) for 10‐20 minutes. Following several washes by ddH_2_O, images were taken and colony numbers were counted.

### 2.7 Cell apoptosis assay

Cell apoptosis was measured by flow cytometry (Guava easyCyte HT, Millipore) using the Annexin V Apoptosis Detection Kit (eBioscience) according to the manufacturer's instruction. Samples with more than 5 × 10^5^ lentivirus‐infected (for 5 days) cells per sample were collected by centrifugation and washed by D‐Hanks buffer (pH = 7.2‐7.4) at 4°C, by 1× binding buffer and then resuspended in 200 μL 1× binding buffer. Then, 10 μL Annexin V‐APC solution was added to the cells, followed by a 10‐15 minutes incubation in the dark. The suspension was diluted with 400‐800 μL 1× binding buffer and analysed with flow cytometer, to determine the percentage of apoptotic cells.

### 2.8 Subcutaneous xenotransplantation of human bladder cancer cells in mice

Twenty‐four‐week‐old BALB/c female nude mice were divided into a normal control (NC) group and a knockdown (KD) group, with 10 mice in each group. 1 × 10^7^ T24 lentivirus‐infected cells carrying shCtrl (NC group) or shPNO1 (KD group) for 7 days were subcutaneously injected into the right arm pit of each mouse. The length (L) and width (W) of tumours were measured from day 24 post‐inoculation and every 3‐5 days until day 36 (tumour volume = 3.14/6 × L × W × W). All mice were then killed by injection of an overdose of 2% pentobarbital sodium followed by cervical vertebra dislocation, and tumours were excised and measured for volume and weight.

Before the mice were killed, tumour sizes were measured by bioluminance live imaging. Each mouse received an intraperitoneal injection of 10 μL per gram bodyweight 15 mg/mL D‐Luciferin Potassium Salt (Sigma‐Aldrich). After 20 minutes, the animals were anesthetized by intraperitoneal injection of 10 μL per gram bodyweight and 0.7% pentobarbital sodium, and examined using Lumina LT In Vivo Imaging Instruments (Perkin Elmer).

### 2.9 DNA microarray and bioinformatics analysis

Total RNA extracted from T24 cells expressing shCtrl or shPNO1 was analysed with the Agilent 2100 Bioanalyzer system (Agilent) and used for preparing amplified RNA (aRNA) with the GeneChip® 3′IVT Express Kit (Affymetrix) according to the manufacturer's instructions. aRNA was purified, and fragmented and hybridized with gene chip probes in a GeneChip Hybridization Oven 645 (Affymetrix). Hybridized microarray was washed and stained with the GeneChip Hybridization Wash and Stain Kit (Affymetrix) and finally scanned with GeneChip Fluidics Station 450 (Affymetrix). Each sample had 3 replicates. The quality of gene chips was controlled to ensure the reliability and repeatability of data. Differentially expressed genes (DEGs) were defined as |FC| > 1.5 (absolute value of fold change) and *P*‐value < .05.

Canonical pathway analysis, upstream regulation analysis, disease and function analysis, regulator effect analysis and gene interaction network analysis were all carried out using the Ingenuity® Pathway Analysis (IPA) software (Qiagen).

### 2.10 Antibody microarray

Antibody microarray was performed using the PathScan® Intracellular Signaling Antibody Array Kit (Chemiluminescent Readout) according to the manufacturer's instructions. T24NC and T24KD cells were washed twice with ice‐cold PBS and lysed with 1× PathScan® Sandwich ELISA Lysis Buffer (Cell Signaling), containing 1 mmol/L PMSF for 2 minutes on ice. Cell lysate was diluted to 0.2‐1.0 mg/mL with the Array Dilution Buffer, which was used in PathScan® Array tests. Arrays were exposed with 5% (v/v) LumiGlo + 5% (v/v) Peroxide in ddH2O for 1‐2 seconds on ChemiScope 5300 (Clinx Science Instruments Co. Ltd).

### 2.11 Statistical analysis

Statistical analysis was performed using the GraphPad Prism 7.0 software. The results were expressed as mean ± standard deviation. The data were analysed initially by *F* test to check the equality of variances. Data with *F*‐value < 0.05 were subjected to two‐tailed Welch's *t* test, and those with *F*‐value > 0.05 were subjected to two‐tailed Student's *t* test. *P* < .05 in the *t* test suggested statistically significant difference.

## 3 RESULTS

### 3.1 Clinicopathological factors associated with PNO1 expression in bladder cancer tissues

**Figure 1:**
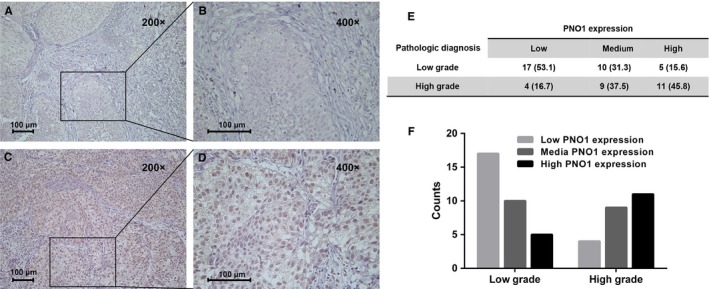
Expression of *PNO1* in bladder cancer. Detection of *PNO1* expression in bladder cancer by immunohistochemistry. (A and B) Low‐grade bladder cancer tissue. (C and D) High‐grade bladder cancer tissue. (E and F) Evaluated *PNO1* expression in 56 bladder urothelial carcinomas

We first evaluated *PNO1* expression in 56 bladder urothelial carcinomas by immunohistochemistry (IHC). The staining of *PNO1* was low or moderate in low‐grade tumours (Figure 1A,B), but strong in high‐grade tumours (Figure 1C,D). Based on the percentage for *PNO1* immune‐positive tumour cells, a score of 1 was given when ≤5% of cells were positive, 2 when 6%‐25%, 3 when 26%‐50% and 4 when ≥ 50% of cells were positive. Staining intensity was scored as 0 (negative), 1 (weak), 2 (moderate) and 3 (strong). Both scores were multiplied, and the resulting score was used to trichotomize PNO1 expression as Low (≤4), Moderate (>5, ≤8) and High (>8). We then analysed the correlation between clinicopathological parameters and *PNO1* expression in the 56 tumour tissue samples (Figure 1E). The expression level of *PNO1*, particularly localized in the nucleus of tumour cells, correlates with high grade (*P* = .0091).

### 3.2 Role of PNO1 in growth and survival of bladder cancer cells

**Figure 2:**
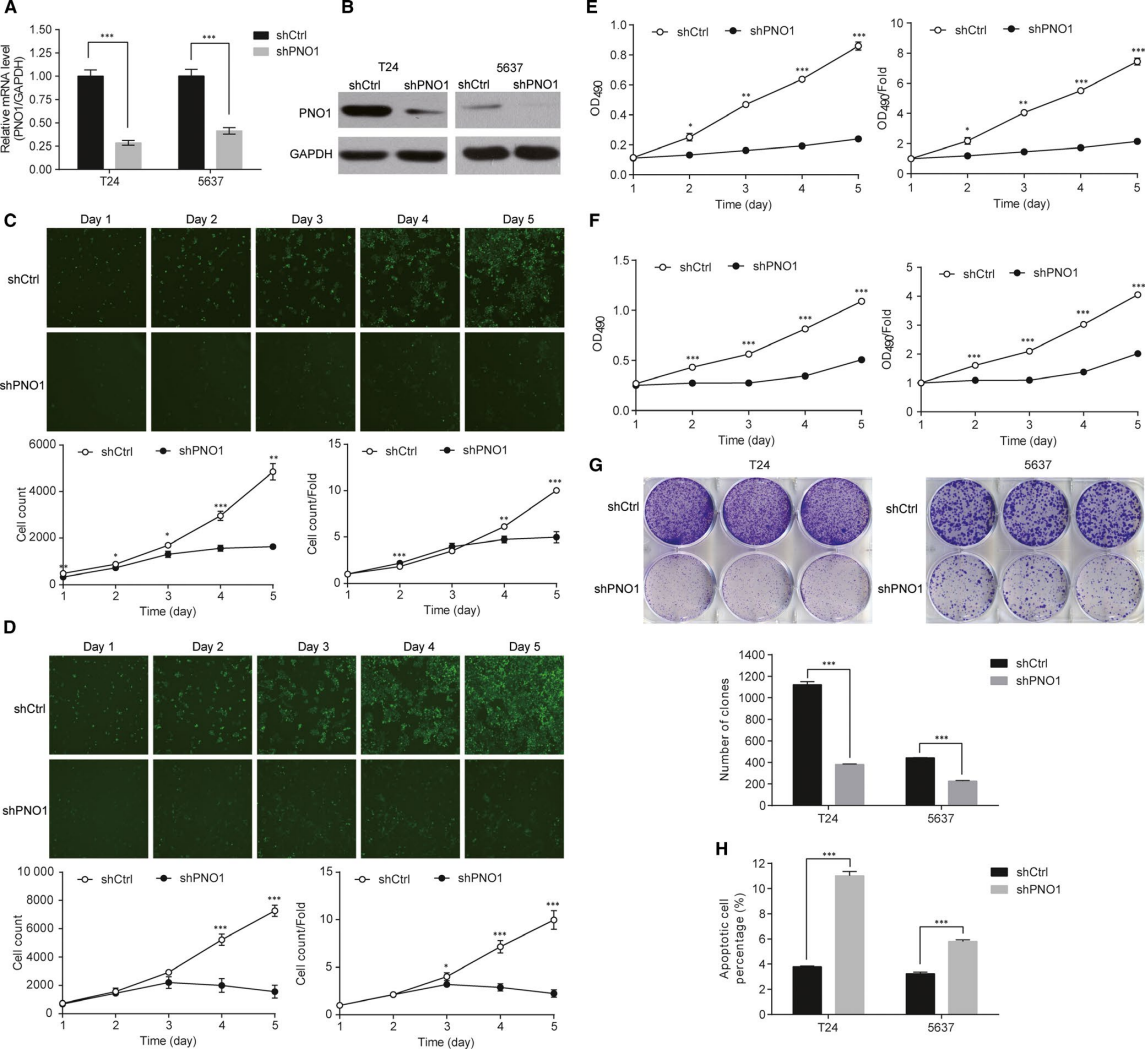
PNO1 knockdown affected the proliferation and survival of T24 and 5637 bladder cells. (A) qRT‐PCR showing significantly reduced mRNA expression level of *PNO1* against *GAPDH* in PNO1 knockdown cells. (B) Western blot showing reduced *PNO1* protein expression in *PNO1* knockdown cells. (C) Growth curves of T24 cells and (D) 5637 cells measured by automated cell counting. (E) Growth curves of T24 cells and (F) 5637 cells measured by MTT assay. (G) Comparison of colony forming ability between control and *PNO1* knockdown cells. (H) Comparison of the percentage of apoptotic cells between control and *PNO1* knockdown cells. *P < .05; **P < .01; ***P < .001

We studied the role of *PNO1* in two bladder cancer cell lines, T24 and 5637. The internal expression level of *PNO1* gene in T24 and 5637 cells was determined by qRT‐PCR. The resulting ΔCt of 7.96 ± 0.181 for T24 and 7.59 ± 0.044 for 5637 suggested high expression of *PNO1* in both bladder cancer cell lines. *PNO1* expression was then knocked down by *PNO1*‐targeting shRNA (shPNO1) via lentivirus, and cells transduced with shCtrl were the control. The knockdown (KD) of *PNO1* in these cell lines was confirmed by qRT‐PCR, which showed 71.5% (*P < *.0001) and 58.6% (*P* = .00023) reduction of *PNO1* mRNA levels in T24 and 5637 cells, respectively (Figure 2A), and by Western blot (Figure 2B).

The effect of *PNO1* KD on the proliferative activity of T24 and 5637 cells was studied using both automated cell counting (Figure 2C,D) and MTT assay (Figure 2E,F). Results showed that, compared with the control cells (shCtrl), the proliferation of *PNO1* KD cells (shPNO1) was significantly attenuated in both cell lines. It was also confirmed that for both cell lines in the KD group, the colonogenesis ability was significantly attenuated (*P < *.001), while cell apoptosis was significantly activated (*P < *.001; Figure 2G,H). These results indicated that *PNO1* was important for the proliferation and survival of bladder cancer cells.

### 3.3 Attenuation tumour growth in vivo after PNO1 knockdown

**Figure 3:**
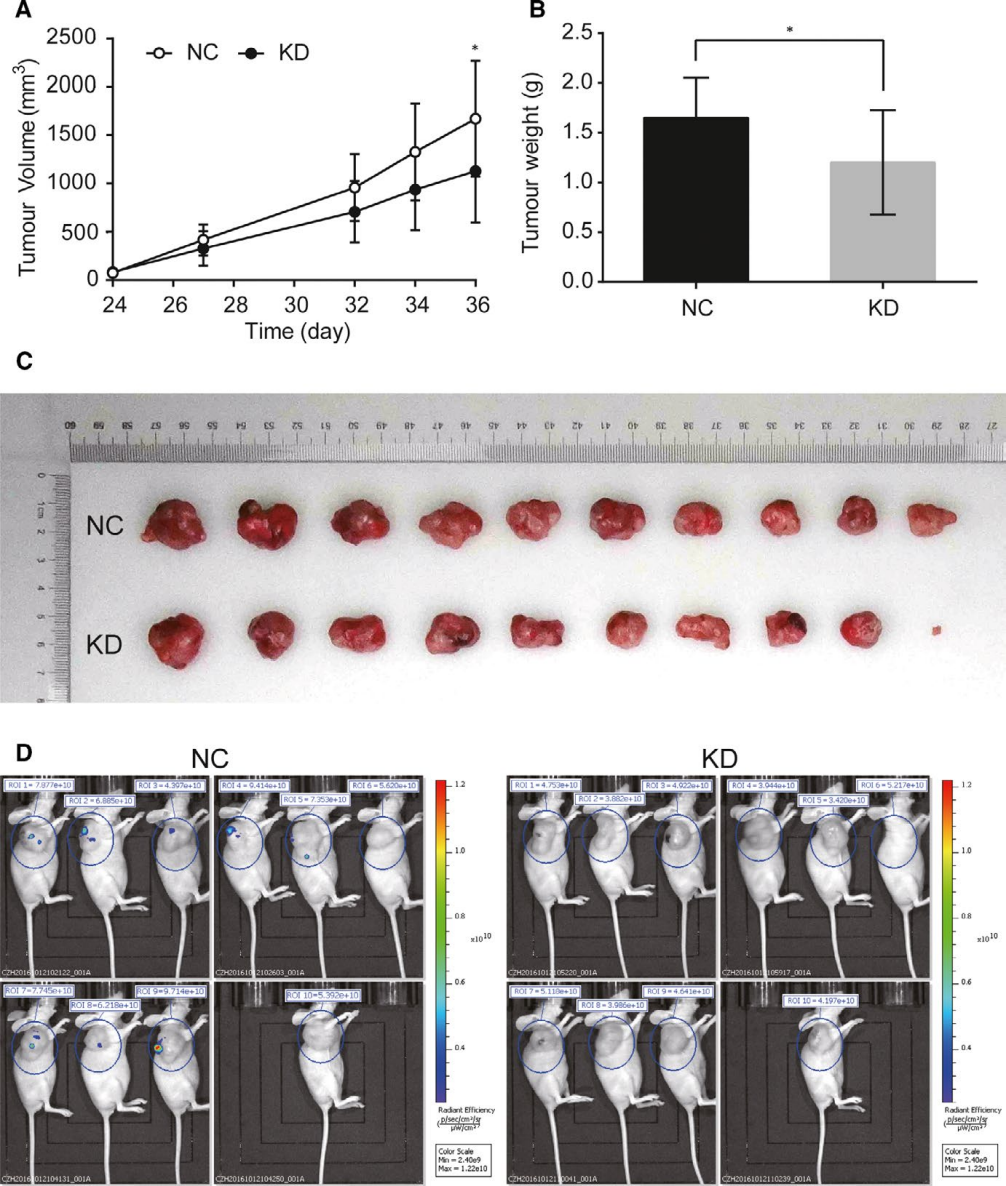
Subcutaneous xenotransplantation of T24 human bladder cancer cells in BALB/c nude mice. (A) Comparison of the volume of xenografts between NC (shCtrl) and KD (shPNO1) group of mice. (B) Comparison of the weight and (C) size of xenografts on day 36 post‐inoculation between NC and KD group of mice. (D) Bioluminance live imaging of mice on day 36 post‐inoculation with total radiant efficiency. ROI, region of interest

To study the function of *PNO1* in bladder cancer in vivo used the BALB/c nude a mouse model; 20 BALB/c female nude mice were subcutaneously inoculated with T24 cells expressing shCtrl (T24NC) or shPNO1 (T24KD), with 10 mice in each group. Size of tumours was measured from day 24 after inoculation (Figure 3A), and mice were killed on day 36 to measure the weight of the xenografts (Figure 3B,C). The final volume and weight of tumours in the NC group was 1670.17 ± 599.48 mm^3^ and 1.652 ± 0.404 g, respectively, compared with 1128.78 ± 529.80 mm^3^ (*P* = .045) and 1.200 ± 0.525 g (*P* = .046) in the KD group. The size of tumours was also measured by bioluminance live imaging before sacrificing the animal, which showed lower total radiant efficiency in KD group of mice (*P < *.001; Figure 3D). Our results confirmed that in a mouse model, the *PNO1* KD cells formed significantly smaller and lighter tumours than the control cells, further confirming the involvement of *PNO1* in tumour growth in vivo.

### 3.4 RNA microarray and IPA analysis of PNO1

#### 3.4.1 RNA microarray identified DEGs in PNO1 KD cells

**Figure 4:**
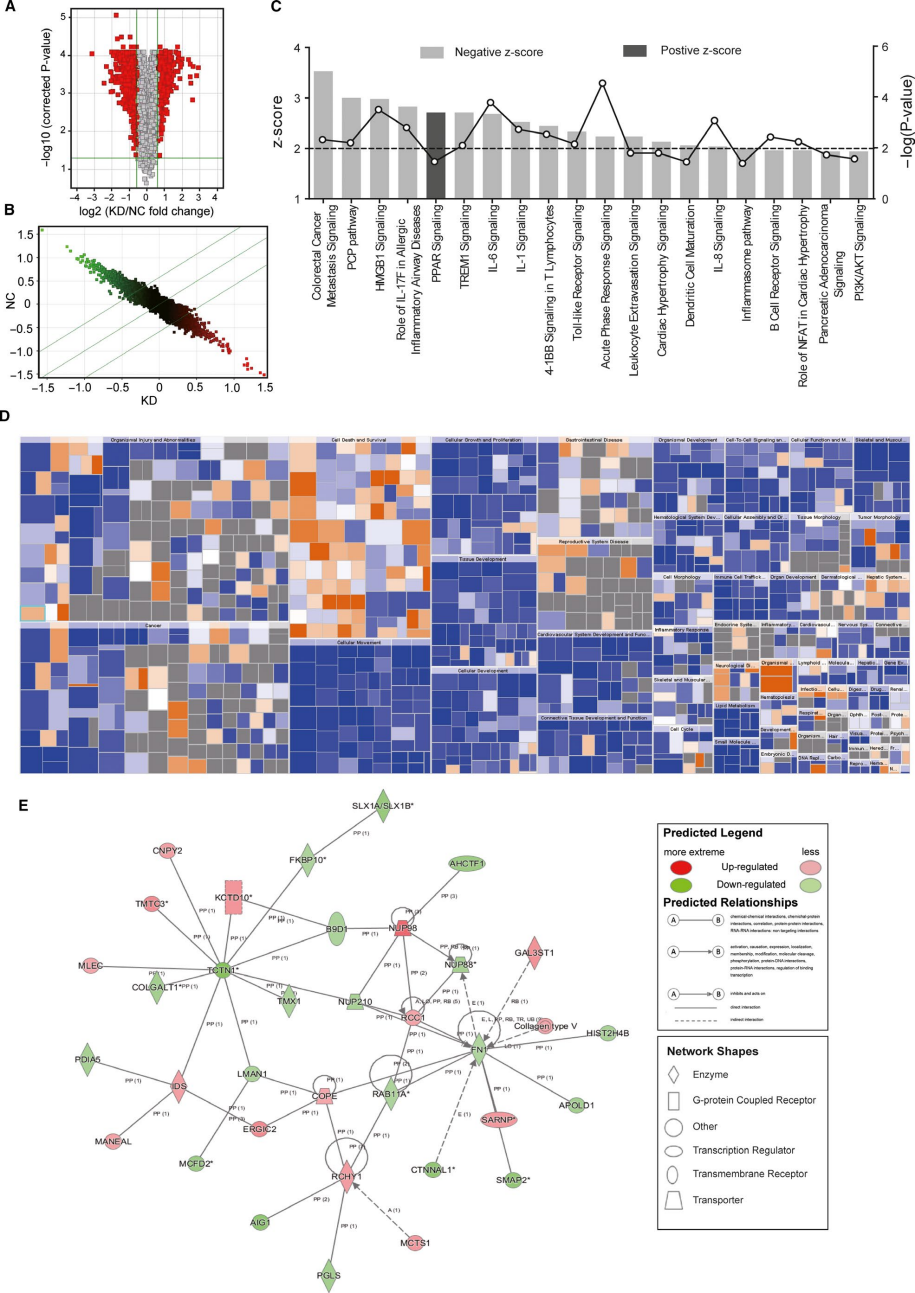
Ingenuity pathway analysis of DNA microarray data of NC and *PNO1* KD T24 cells. (A) Volcano diagram demonstrating the distribution of DEGs between NC and KD group. Red dots represented probes with |FC| > 1.5 and *P* < .05. (B) Scatter diagram showing the distribution of signals in the Cartesian coordinate system. Dots above the top green line were down‐regulated probes in KD group comparing to NC. Dots below the bottom green line were up‐regulated probes in KD group comparing to NC. (C) Signal pathway histogram showing the enrichment of DEGs in canonical signalling pathways. *Z*‐score (bars) reflected the extent to which the pathway was suppressed (*Z*‐score < 0) or activated (*Z*‐score > 0). |*Z*‐score| > 2 was the threshold of significance. −log(*P*‐value; dots and lines) reflected the likelihood of the involvement of this pathway. (D) Heat map showing the participation of DEGs in diseases and physiological functions. Orange, *Z*‐score > 0; blue, *Z*‐score < 0; and grey, no *Z*‐score. (E) Interaction network diagram of molecules within the top‐scored network

Gene expression in T24KD and T24NC cells was analysed by RNA microarray. A total of 1543 DEGs were identified, including 675 up‐regulated genes and 868 down‐regulated genes in T24KD cells compared with T24NC cells, with *PNO1* down‐regulated by 3.42‐fold (*P* = 2.97 × 10^−5^). Distribution of DEGs between groups is shown in Figure 4A,B. RNA microarray data were subjected to IPA analysis.

#### 3.4.2 Canonical pathway analysis

Sixteen significantly altered canonical signalling and metabolic pathways correlated with DEGs (−log *P* > 1.3 and |*Z*‐score| > 2) and were summarized by IPA (Figure 4C, Table 1). The Acute‐phase response signalling pathway was the most significantly enriched and down‐regulated pathway involving the identified DEGs (−log *P* = 4.54), whose signalling networks are shown in Figure S1 and Figure S2. Colorectal cancer metastasis signalling was the most down‐regulated pathway (*Z*‐score = 3.53). Peroxisome proliferator‐activated receptors (PPAR) signalling was the only up‐regulated pathway.

**Table 1:** List of significantly up‐ or down‐regulated regulated canonical pathways that were significantly related to differentially expressed genes (DEGs) between NC and PNO1 KD T24 cells

Ingenuity canonical pathways	−log(*P*‐value)	*Z*‐score
Colorectal Cancer Metastasis Signalling	2.32	−3.53
PCP pathway	2.19	−3
HMGB1 Signalling	3.5	−2.982
Role of IL‐17F in Allergic Inflammatory Airway Diseases	2.79	−2.828
TREM1 Signalling	2.09	−2.714
IL‐6 Signalling	3.78	−2.683
IL‐1 Signalling	2.72	−2.53
4‐1BB Signalling in T Lymphocytes	2.53	−2.449
Toll‐like Receptor Signalling	2.14	−2.333
Acute‐Phase Response Signalling	4.54	−2.236
Leucocyte Extravasation Signalling	1.79	−2.236
Cardiac Hypertrophy Signalling	1.79	−2.132
Dendritic Cell Maturation	1.44	−2.065
IL‐8 Signalling	3.07	−2.041
Inflammasome pathway	1.38	−2
PPAR Signalling	1.45	2.714

Note

−log(*P*‐value) > 1.3, which meant *P* < .05, represented significant correlation between DEGs and the pathway. *Z*‐score > 2 suggested significant activation of the pathway while *Z*‐score < −2 suggested significant repression of the pathway.

#### 3.4.3 Upstream regulation analysis

The upstream regulating genes of DEGs were analysed. One hundred and fifty‐eight genes were predicted to significantly up‐regulate the DEGs, among which the kinase inhibitor chemical U0126 was the upstream regulator (Activation *Z*‐score = 5.905, *P* = 1.45 × 10^−11^) to be activated most strongly, along with 48 unanimously activated genes. Three hundred and fifty‐nine genes were predicted to significantly down‐regulate the DEGs, among which phorbol myristate acetate was the upstream regulator (Activation *Z*‐score = −6.063, *P* = 1.90 × 10^−9^) to be inhibited most strongly, compared with 80 unanimously inhibited genes. The regulation network of phorbol myristate acetate and its downstream genes are shown in Figure S3.

#### 3.4.4 Disease and function analysis

The inhibition or activation of diseases and physiological functions by up‐ or down‐regulated DEGs was analysed and plotted in Figure 4C. The 6 activated functions in T24KD cells were organismal death and morbidity, or mortality of the organismal survival category, apoptosis of lymphoma cell lines, anoikis, cell death of breast cell lines and apoptosis of lung cells of the Cell Death and Survival category. The suppressive categories in T24KD cells included cellular movement (chemotaxis, homing of cells, migration of cells, cell movement, cell movement of tumour cell lines, migration, invasion of tumour cell lines, *etc*), inflammatory response, lipid metabolism, small molecule biochemistry (synthesis of eicosanoid, biosynthesis of polyunsaturated fatty acids, synthesis of fatty acid, *etc*), cancer, organismal injury and abnormalities (growth of malignant tumour, growth of tumour, proliferation of tumour cells, neoplasia of tumour cell lines, *etc*), cardiovascular system development and function, organismal development, cell‐to‐cell signalling, interaction, *etc.*

#### 3.4.5 Regulator effect analysis

The possible routes of DEGs participating in the upstream regulation network and downstream functions were analysed. The result predicted lysophosphatidic acid had down‐regulated cell movement of tumour cell lines through *CCND1*, *CXCL2*, *CXCL3*, *CXCL8*, *CYR61*, *EGFR*, *EGR1*, *F3*, *IL1B*, *IL6*, *KLF5*, *PLAU*, *PLAUR*, *PTGS2*, *S1PR3*, *SERPINE1*, *TNF* and *WNT5A* with the highest consistency score (Figure S4).

#### 3.4.6 Interaction network among DEGs

The interaction networks among all DEGs were also analysed, and results showed that the top diseases and functions significantly correlated with interacting molecules within the network were as follows: haematological disease, hereditary disorder, organismal injury and abnormalities, which involved 34 DEGs (Table 2). The interaction of molecules within the top‐scored network was presented in Figure 4E.

**Table 2:** List of interaction networks among DEGs ranked according to the significance level, which reflected the number of DEGs included

Score	Focus molecules	Top diseases and functions
45	34	Haematological Disease, Hereditary Disorder, Organismal Injury and Abnormalities
40	33	RNA Post‐Transcriptional Modification, Hereditary Disorder, Ophthalmic Disease
40	32	Cancer, Organismal Injury and Abnormalities, Reproductive System Disease
38	31	Cellular Assembly and Organization, DNA Replication, Recombination, and Repair, Cellular Movement
38	31	Cardiac Arteriopathy, Cardiovascular Disease, Organismal Injury and Abnormalities
38	31	Cellular Assembly and Organization, Developmental Disorder, Hereditary Disorder
36	30	Lipid Metabolism, Molecular Transport, Small Molecule Biochemistry
36	30	Amino Acid Metabolism, Drug Metabolism, Small Molecule Biochemistry
36	31	Cancer, Cellular Assembly and Organization, Cellular Development
34	29	Metabolic Disease, Neurological Disease, Organismal Injury and Abnormalities
34	29	Hereditary Disorder, Ophthalmic Disease, Organismal Injury and Abnormalities
34	29	Cancer, Cell Death and Survival, Organismal Injury and Abnormalities
34	29	Cellular Development, Cellular Growth and Proliferation, Embryonic Development
32	28	Cellular Assembly and Organization, Cellular Function and Maintenance, Connective Tissue Disorders
32	28	Developmental Disorder, Hereditary Disorder, Metabolic Disease
32	28	Developmental Disorder, Hereditary Disorder, Ophthalmic Disease
31	29	Post‐Translational Modification, Cancer, Cell Cycle
30	28	Cellular Development, Cellular Growth and Proliferation, Haematopoiesis
30	27	Post‐Translational Modification, Cell‐To‐Cell Signalling and Interaction, Hair and Skin Development and Function
28	26	Nucleic Acid Metabolism, Small Molecule Biochemistry, Cancer
26	25	Cellular Compromise, Cellular Assembly and Organization, Cardiovascular Disease
24	24	Cell Death and Survival, Cellular Movement, Cardiovascular System Development and Function
23	24	Cellular Movement, Cellular Development, Cellular Growth and Proliferation
23	23	Cellular Development, Cellular Growth and Proliferation, Embryonic Development
22	23	Developmental Disorder, Hereditary Disorder, Neurological Disease

Note

Focus Molecules showed the total number of DEGs within the network.

### 3.5 PNO1‐regulated gene expression

**Figure 5:**
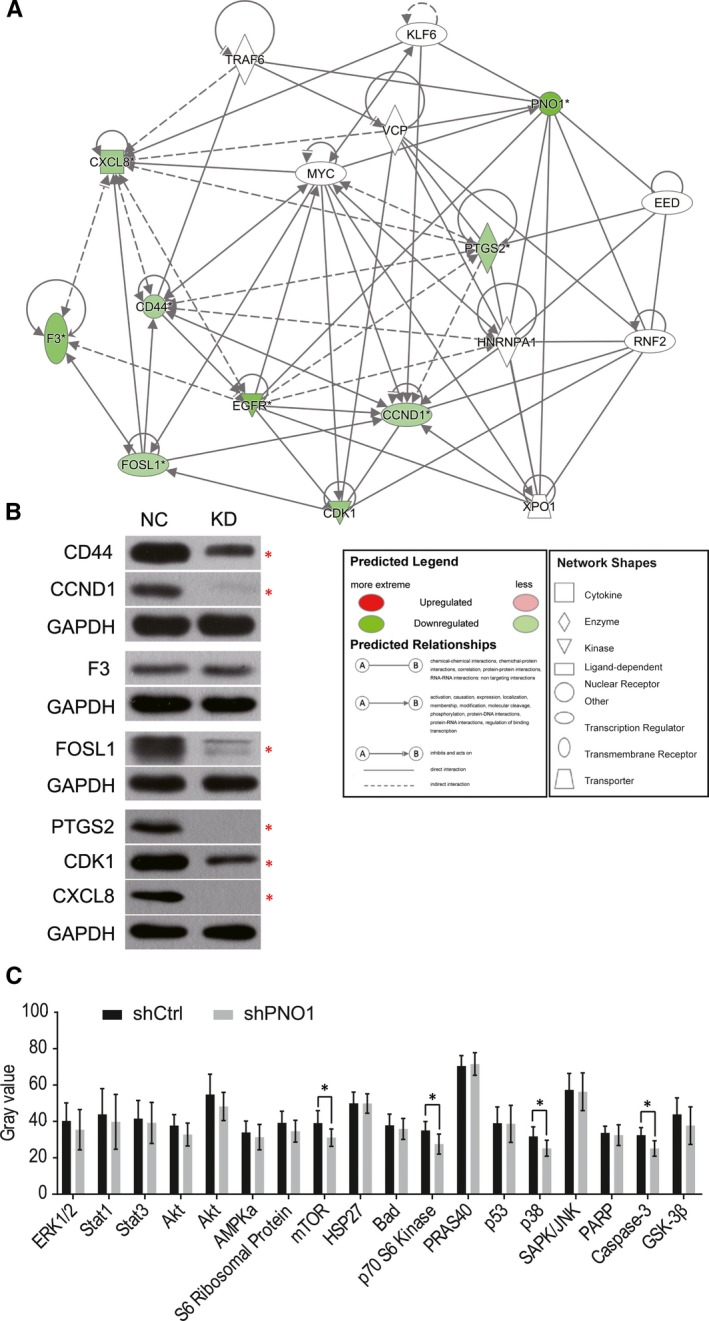
*PNO1* downstream gene expression. (A) Interaction network diagram of *PNO1* with selected downstream genes. (B) Western blot of *PNO1* downstream genes in T24NC and T24KD cells. (C) Comparison of the expression of 18 key node proteins of intracellular signalling pathways that are closely associated with diseases in T24NC and T24KD cells. *P < .05

Based on the above informatics analysis, we chose *PNO1* and some of its downstream genes (*CD44*, *PTGS2*, *CCND1*, *CDK1*, *F3*, *CXCL8* and *FOSL1)* for interaction analysis, and the gene interaction network is shown in Figure 5A. The protein expression of these genes was further confirmed by Western blot in T24NC and T24KD cells (Figure 5B). Apart from F3, the expression of all genes was significantly reduced as a result of *PNO1* knockdown, which was consistent with the microarray result.

In addition, the effect of *PNO1* on 18 key node proteins of intracellular signalling pathways that are closely associated with diseases was studied by antibody array. Results showed that, among these proteins, mTOR, p70 S6 kinase, p38 and Caspase‐3 were significantly down‐regulated in *PNO1* KD cells (Figure 5C).

## 4 DISCUSSION

Previously PNO1 was well known as a RNA‐binding protein in humans, and its ortholog PNO1 was reported to participate in ribosome and proteasome biogenesis in yeasts. Wang had generated PNO1 gene knockout (KO) and PNO1 overexpressing transgenic (Tg) mice lineages. Homozygous PNO1 KO lineage caused early lethality in mouse embryos, while heterozygous mice with 50% of normal Pno1 mRNA concentration and Tg lineage were fertile and showed no obvious anomalies.^6^

Deregulation of cell proliferation and evasion of apoptosis are two hallmarks of cancer cells. Our study is the first report to show elevated expression of *PNO1* in UBC tissues. *PNO1* KD inhibited the cell proliferation and colonogenesis ability, promoted cell apoptosis and reduced the tumorigenic ability of UBC, suggesting the pro‐tumour role of *PNO1* in UBC.

As a result of *PNO1* KD, 1543 genes were found differentially expressed in T24 cells, which were involved in a number of signalling pathways, biological processes, regulatory cascades and interactive networks. In accordance with the cellular functional study results, IPA analysis confirmed that several apoptosis‐related functions were enhanced in *PNO1* KD cells, while neoplasia, growth and proliferation of tumour cells were inhibited. Besides this, the functions of chemotaxis, cell movement, and migration and invasion of tumour cells were also down‐regulated in *PNO1* KD cells, indicating that *PNO1* might also participate in the metastasis of cancer cells.

IPA analysis retrieved 16 significantly altered canonical pathways from the DEGs, among which the PPAR signalling was the only up‐regulated pathway after KD of *PNO1*. As one of the three peroxisome proliferator‐activated receptors, PPARγ is a ligand‐activated transcription factor of the nuclear hormone receptor superfamily, which have a contradictory role on tumorigenicity, in addition to its role in glucose and lipid metabolism and inflammation.^10^ A number of PPARγ ligands were reported to bear a potential of increased risk of UBC.^11^, ^12^ Previous study suggested that loss of PPARγ may be an important step in the progression of UBC.^13^ The natural ligand of PPARγ, 15d‐PGJ(2), could inhibit the proliferation and induce the apoptosis of T24 and 5637 cells, through induction of the production of reactive oxygen species (ROS).^14^ However, another study reported dramatically increased amplification of PPARγ in UBC tissues, especially those with lymph node metastasis and that PPARγ played an important role in 5673 cell migration and invasion.^15^ This controversy in PPARγ function reflected the complexity of PPAR signal modulating mechanism in UBC.

Among the 15 significantly down‐regulated canonical pathways in T24KD cells, the vast majority were immunoreaction‐related pathways, involving important components of both the innate immune response (HMGB1, IL‐7F, TREM1, IL‐1, Toll‐like Receptor, leucocyte extravasation, dendritic cell maturation, IL‐8, inflammasome signalling and acute‐phase response) and adaptive immune response (IL‐6 and 4‐1BB signalling). The PCP pathway participates in the regulation of the cytoskeleton. Therefore, its down‐regulation in *PNO1* KD cells further indicated the role of *PNO1* in the migration of UBC.

The acute‐phase response was the most significantly enriched down‐regulated pathway involving the identified DEGs, which is characterized by alteration in the concentrations of a number of plasma proteins produced by the liver, in response to cytokines including IL‐1, IL‐6, IL‐8, TNFα, *etc* during inflammatory response.^16^ The acute‐phase proteins (APPs), C‐reactive protein (CRP) and orosomucoid (ORM) were reported to be important prognostic or diagnostic biomarkers for UBC.^17^, ^18^ Comparing the identified activation/inhibition status of proteins in the acute‐phase response signalling based on our test result (Figure S1) and that reported by current literature (Figure S2), it was noted that the positive APPs, serum amyloid A (SAA) and ceruloplasmin (CP) were activated, while the negative APP, suppressor of cytokine signalling proteins (SOCS), was inhibited in the down‐regulated acute‐phase response signalling pathway, which was abnormal. This was either due to experimental errors or indicated potential new pathways downstream of *PNO1*.

The expression of 6 proteins, CD44, PTGS2, cyclin D1 (encoded by *CCND1*), CDK1, IL‐8 (encoded by *CXCL8*) and FRA1 (encoded by *FOSL1*), was confirmed to be down‐regulated in *PNO1* KD cells. CD44 antigen is a cell‐surface glycoprotein involved in cell–cell interactions, cell adhesion and cell migration. It is stimulated by IL‐6 and was implicated with higher clinical stage, radio‐resistance, cancer stem cell‐like property and resistance to apoptosis in UBC.^19^, ^20^, ^21^

Prostaglandin‐endoperoxide synthase 2 (PTGS2) is also known as cyclooxygenase‐2 (COX‐2),^22^ which was one of the two isoforms of cyclooxygenases. Cyclooxygenases catalyse the initial step in the formation of prostaglandins (PGs), which are involved in various inflammatory cell processes including carcinogenesis. As PTGS1 was predominantly expressed in urothelium while UBC cells exhibited PTGS2 overexpression,^23^ the transition between PTGS isoforms might contribute to the tumorigenesis of UBC. PTGS2 activation might also be involved in inflammation‐mediated cancer stem cell proliferation during bladder carcinogenesis.^24^ Cyclin D1 (CCND1) is involved in regulating the cell cycle progression and growth factor signalling, making it a possible oncogene. One of its two isoforms, cyclin D1b is important for the malignant phenotypes of human UBC cells via suppression of apoptosis, induction of cancer cell stemness and epithelial–mesenchymal transition.^25^ Altered expression of cyclin D1 was reported to be associated with lymph node metastasis and risk of recurrence of UBC.^26^ As a binding partner of cyclins, cyclin‐dependent kinase 1 (CDK1) is also a key player in cell cycle regulation. Fos‐related antigen 1 (FRA1) encodes a leucine zipper protein that can dimerize with proteins of the JUN family. FRA1 promotes cancer growth through AKT and enhances cancer cell migration through JNK/c‐Jun in squamous cell carcinoma.^27^ IL‐8 induces chemotaxis in neutrophils and other granulocytes, as well as phagocytosis. Therefore, it was possible that the down‐regulated chemotaxis function, as previously mentioned, might be caused by inhibition of IL‐8 expression.

This study also revealed a down‐regulation of mTOR, p70 S6 kinase, p38 and caspase‐3 proteins in *PNO1* KD cells, suggesting the association of *PNO1* with several intracellular signalling pathways. mTOR and p70 S6 kinase are both key components of the mTOR signalling pathway, which promotes cell growth and proliferation in eukaryotic cells.^28^ Altered mTOR pathway activity has been noted in a variety of human tumours, including urothelial carcinoma.^29^ mTOR pathway activation was reported to be involved in UBC tumorigenesis and was a predictor of cancer progression and poor survival.^30^ p38 mitogen‐activated protein kinases belong to the MAPK family and are activated by stress stimuli, such as cytokines, ultraviolet irradiation, heat shock and osmotic shock. The activation of p38 MAPKs has been reported to contribute to the epithelial‐mesenchymal transition of cells in the primary tumour, to the acquisition of invasion and migrating capabilities and to the extravasation of migrating tumour cells, while p38 MAPK inhibition has been correlated with the resistance to anoikis.^31^ Caspase‐3 belongs to the caspase family and plays an indispensable role in the execution‐phase of cell apoptosis.^32^ However, the significantly increased apoptotic activity in T24KD cells that was observed contradicted the down‐regulation of caspase‐3. We suspected that this might be caused by experimental errors.

This is the first time the cellular functions and clinical significance of *PNO1* in UBC was investigated, and its molecular mechanism explored through microarrays and bioinformatics analysis. Our findings confirm that *PNO1* participates in promoting proliferation and colonogenesis, while reducing apoptosis of UBC cells, and is also predicted to be associated with the migration and metastasis of UBC *PNO1* KD attenuated the tumorigenesis ability of UBC in mouse. *PNO1* KD led to the altered expression of 1543 genes that are involved in a number of signalling pathways, biological functions and regulation networks. CD44, PTGS2, cyclin D1, CDK1, IL‐8, FRA1, as well as mTOR, p70 S6 kinase, p38, and caspase‐3 proteins were all down‐regulated in *PNO1* KD cells, suggesting the involvement of *PNO1* in inflammatory responses, cell cycle regulation, chemotaxis, cell growth and proliferation, apoptosis, cell migration and invasiveness. This study will add to our understanding of the molecular mechanism of UBC and hopefully provide novel targets for individualized cancer therapy.

## CONFLICT OF INTEREST

The authors confirm that there are no conflict of interests.

## AUTHOR CONTRIBUTION

Chunhua Lin and Jitao Wu designed and conducted the experiments and analysed data. Wenting Wang was involved in the design, animal experiment and drafting of the manuscript. Hejia Yuan and Youyi Lu collected the data of patients with bladder cancer. Jiahui Wang performed Western blot and participated in the animal experiment. Hejia Yuan and Fan Feng were responsible for cell culture. Zhe Zhu and Jitao Wu wrote and revised the manuscript.

## Data Availability

The data that support the findings of this study are available from the corresponding author upon reasonable request.

## References

[1] Ferlay J , Soerjomataram I , Dikshit R , et al. Cancer incidence and mortality worldwide: sources, methods and major patterns in GLOBOCAN 2012. Int J Cancer. 2015;136:E359‐E386. 25220842 10.1002/ijc.29210

[2] Siegel RL , Miller KD , Jemal A . Cancer statistics, 2018. CA: Cancer J Clin. 2018;68(1):7‐30. 29313949 10.3322/caac.21442

[3] Chen W , Zheng R , Baade PD , et al. Cancer statistics in China, 2015. CA: Cancer J Clin. 2016;66(2):115‐132. 26808342 10.3322/caac.21338

[4] Piao XM , Byun YJ , Kim WJ , Kim J . Unmasking molecular profiles of bladder cancer. Invest Clin Urol. 2018;59(2):72‐82. 10.4111/icu.2018.59.2.72 PMC5840121 29520382

[5] Zhou GJ , Zhang Y , Wang J , et al. Cloning and characterization of a novel human RNA binding protein gene PNO1. DNA Seq. 2004;15:219‐224. 15497447 10.1080/10425170410001702159

[6] Wang X , Wu T , Hu Y , et al. Pno1 tissue‐specific expression and its functions related to the immune responses and proteasome activities. PLoS ONE. 2012;7:e46093. 23029399 10.1371/journal.pone.0046093 PMC3461026

[7] Vanrobays E , Gelugne JP , Caizergues‐Ferrer M , Lafontaine DL . Dim2p, a KH‐domain protein required for small ribosomal subunit synthesis. RNA (New York, NY). 2004;10:645‐656. 10.1261/rna.5162204 PMC1370555 15037774

[8] Tone Y , Toh EA . Nob1p is required for biogenesis of the 26S proteasome and degraded upon its maturation in *Saccharomyces cerevisiae* . Genes Dev. 2002;16:3142‐3157. 12502737 10.1101/gad.1025602 PMC187499

[9] Lin C , Wang J , Wang Y , et al. GRP78 participates in PCA3‐regulated prostate cancer progression. Anticancer Res. 2017;37:4303‐4310. 28739722 10.21873/anticanres.11823

[10] Cariou B , Charbonnel B , Staels B . Thiazolidinediones and PPARgamma agonists: time for a reassessment. Trends Endocrinol Metab: TEM. 2012;23:205‐215. 22513163 10.1016/j.tem.2012.03.001

[11] Yang SL , Wang JJ , Chen M , et al. Pioglitazone use and risk of bladder cancer: an in vitro study. Int J Med Sci. 2018;15:228‐237. 29483814 10.7150/ijms.22408 PMC5820852

[12] Yousefnia S , Momenzadeh S , Seyed Forootan F , Ghaedi K , Nasr Esfahani MH . The influence of peroxisome proliferator‐activated receptor gamma (PPARgamma) ligands on cancer cell tumorigenicity. Gene. 2018;649:14‐22. 29369787 10.1016/j.gene.2018.01.018

[13] Nakashiro KI , Hayashi Y , Kita A , et al. Role of peroxisome proliferator‐activated receptor gamma and its ligands in non‐neoplastic and neoplastic human urothelial cells. Am J Pathol. 2001;159:591‐597. 11485917 10.1016/s0002-9440(10)61730-0 PMC1850548

[14] Wang Y , Tan H , Xu D , et al. The combinatory effects of PPAR‐gamma agonist and survivin inhibition on the cancer stem‐like phenotype and cell proliferation in bladder cancer cells. Int J Mol Med. 2014;34:262‐268. 24820432 10.3892/ijmm.2014.1774

[15] Yang DR , Lin SJ , Ding XF , et al. Higher expression of peroxisome proliferator‐activated receptor gamma or its activation by agonist thiazolidinedione‐rosiglitazone promotes bladder cancer cell migration and invasion. Urology. 2013;81(1109):e1‐e6. 23522297 10.1016/j.urology.2012.12.027

[16] Davalieva K , Kiprijanovska S , Maleva Kostovska I , et al. Comparative proteomics analysis of urine reveals down‐regulation of acute phase response signaling and LXR/RXR activation pathways in prostate cancer. Proteomes. 2017;6(1):1. 10.3390/proteomes6010001 PMC5874760 29286311

[17] Mbeutcha A , Shariat SF , Rieken M , et al. Prognostic significance of markers of systemic inflammatory response in patients with non‐muscle‐invasive bladder cancer. Urol Oncol. 2016;34(11):483.e17‐483.e24. 10.1016/j.urolonc.2016.05.013 27646875

[18] Irmak S , Tilki D , Heukeshoven J , et al. Stage‐dependent increase of orosomucoid and zinc‐alpha2‐glycoprotein in urinary bladder cancer. Proteomics. 2005;5:4296‐4304. 16196100 10.1002/pmic.200402005

[19] Oldenburg D , Ru Y , Weinhaus B , Cash S , Theodorescu D , Guin S . CD44 and RHAMM are essential for rapid growth of bladder cancer driven by loss of Glycogen Debranching Enzyme (AGL). BMC Cancer. 2016;16:713. 27595989 10.1186/s12885-016-2756-5 PMC5011830

[20] Yikilmaz TN , Dirim A , Ayva ES , Ozdemir H , Ozkardes H . Clinical use of tumor markers for the detection and prognosis of bladder carcinoma: a comparison of CD44, cytokeratin 20 and survivin. Urol J. 2016;13:2677‐2683. 27351322

[21] Wu CT , Lin WY , Chang YH , Chen WC , Chen MF . Impact of CD44 expression on radiation response for bladder cancer. J Cancer. 2017;8:1137‐1144. 28607587 10.7150/jca.18297 PMC5463427

[22] Hla T , Neilson K . Human cyclooxygenase‐2 cDNA. Proc Natl Acad Sci. 1992;89(16):7384‐7388. 1380156 10.1073/pnas.89.16.7384 PMC49714

[23] Bostrom PJ , Aaltonen V , Soderstrom KO , Uotila P , Laato M . Expression of cyclooxygenase‐1 and ‐2 in urinary bladder carcinomas in vivo and in vitro and prostaglandin E2 synthesis in cultured bladder cancer cells. Pathology. 2001;33:469‐474. 11827414 10.1080/00313020120083188

[24] Thanan R , Murata M , Ma N , et al. Nuclear localization of COX‐2 in relation to the expression of stemness markers in urinary bladder cancer. Mediators Inflamm. 2012;2012:165879. 22577245 10.1155/2012/165879 PMC3337674

[25] Kim CJ , Terado T , Tambe Y , et al. Anti‐oncogenic activities of cyclin D1b siRNA on human bladder cancer cells via induction of apoptosis and suppression of cancer cell stemness and invasiveness. Int J Oncol. 2018;52:231‐240. 29115414 10.3892/ijo.2017.4194

[26] Kopparapu PK , Boorjian SA , Robinson BD , et al. Expression of cyclin d1 and its association with disease characteristics in bladder cancer. Anticancer Res. 2013;33:5235‐5242. 24324055 PMC4122540

[27] Zhang X , Wu J , Luo S , Lechler T , Zhang JY . FRA1 promotes squamous cell carcinoma growth and metastasis through distinct AKT and c‐Jun dependent mechanisms. Oncotarget. 2016;7:34371‐34383. 27144339 10.18632/oncotarget.9110 PMC5085162

[28] Foster KG , Fingar DC . Mammalian target of rapamycin (mTOR): conducting the cellular signaling symphony. J Biol Chem. 2010;285:14071‐14077. 20231296 10.1074/jbc.R109.094003 PMC2863215

[29] Ching CB , Hansel DE . Expanding therapeutic targets in bladder cancer: the PI3K/Akt/mTOR pathway. Lab Invest. 2010;90(10):1406‐1414. 20661228 10.1038/labinvest.2010.133

[30] Park SJ , Lee TJ , Chang IH . Role of the mTOR pathway in the progression and recurrence of bladder cancer: an immunohistochemical tissue microarray study. Kor J Urol. 2011;52:466‐473. 10.4111/kju.2011.52.7.466 PMC3151634 21860767

[31] Koul HK , Pal M , Koul S . Role of p38 MAP kinase signal transduction in solid tumors. Genes Cancer. 2013;4:342‐359. 24349632 10.1177/1947601913507951 PMC3863344

[32] Martin SJ , Green DR . Protease activation during apoptosis: death by a thousand cuts? Cell. 1995;82:349‐352. 7634323 10.1016/0092-8674(95)90422-0

