# Supplementary figures and images for: Importance of PNO1 for growth and survival of urinary bladder carcinoma: Role in core‐regulatory circuitry
Source: J Cell Mol Med. 2019 Dec 4;24(2):1504–15. doi: 10.1111/jcmm.14835 (PMC6991670; doi:10.1111/jcmm.14835)

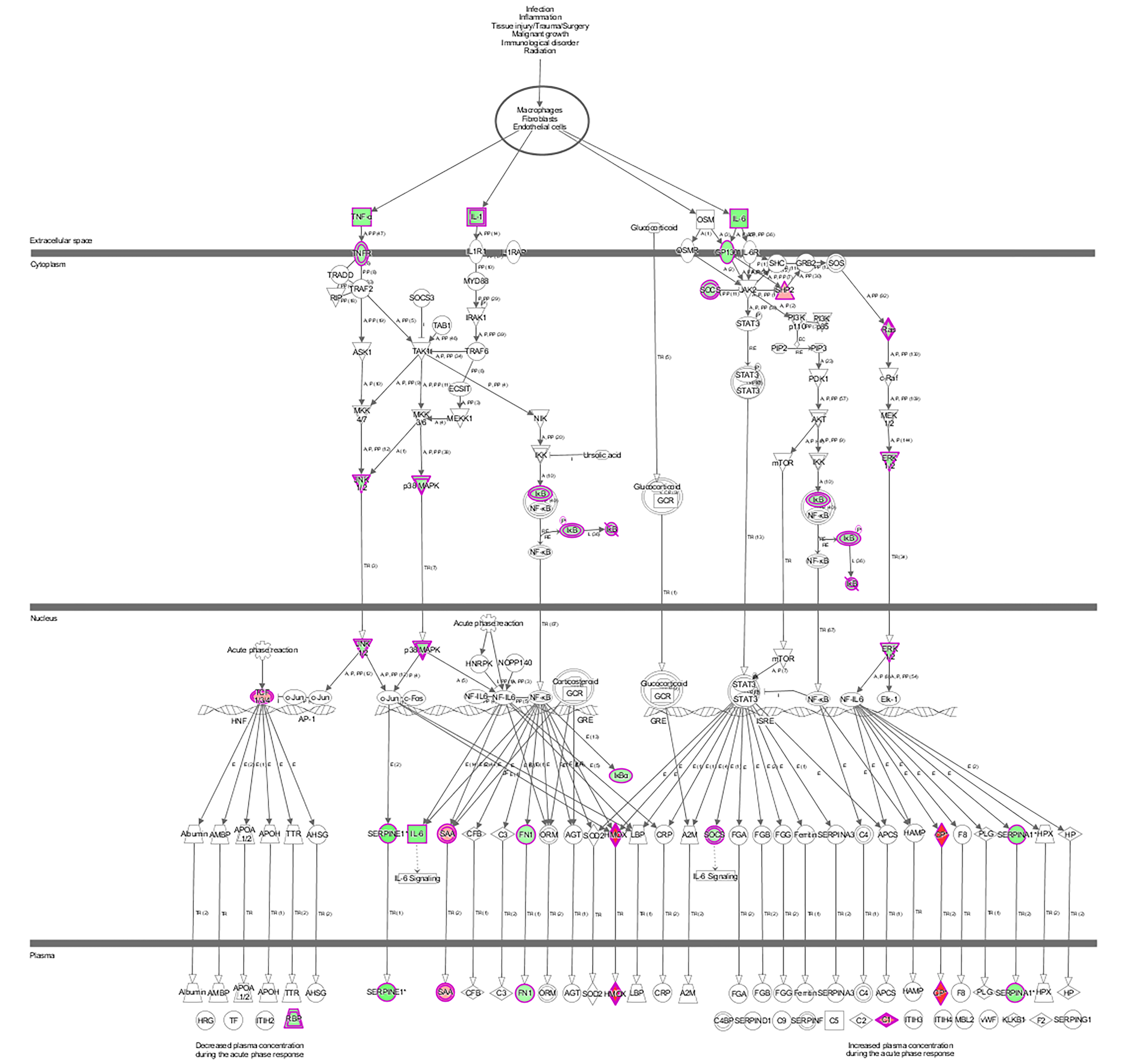

Supplement: Supplementary file 1 [file JCMM-24-1504-s001.png]

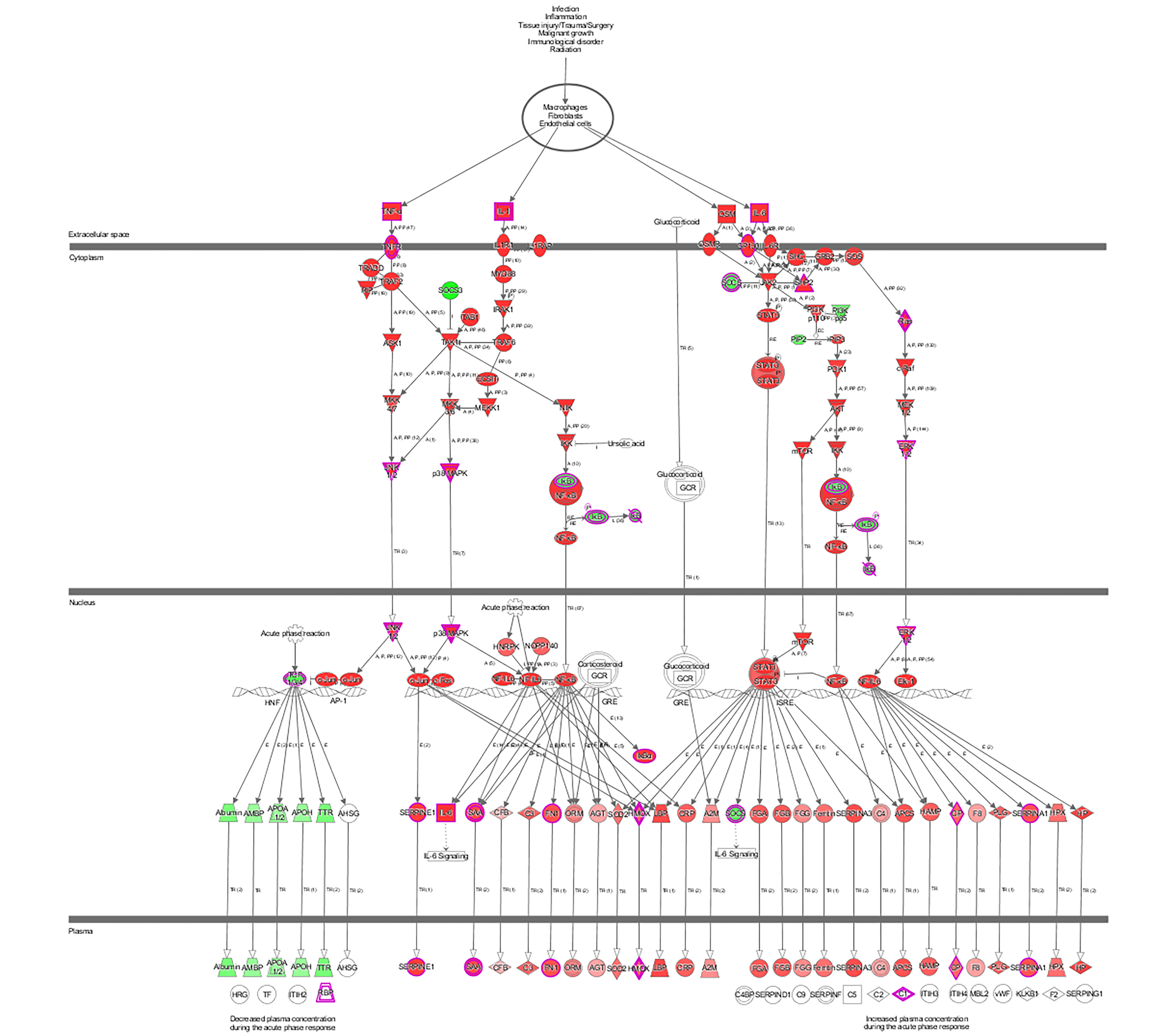

Supplement: Supplementary file 2 [file JCMM-24-1504-s002.png]

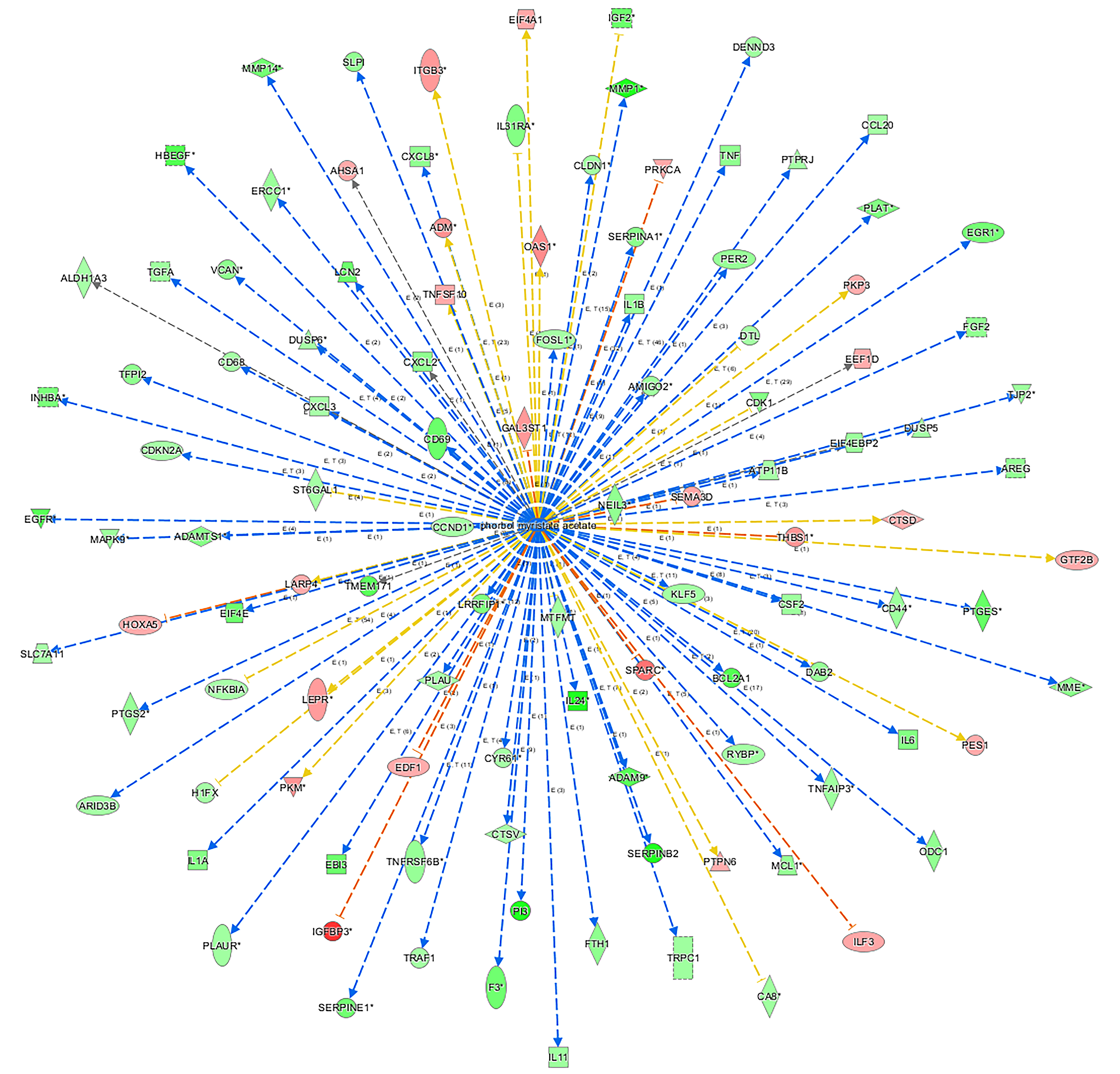

Supplement: Supplementary file 3 [file JCMM-24-1504-s003.png]

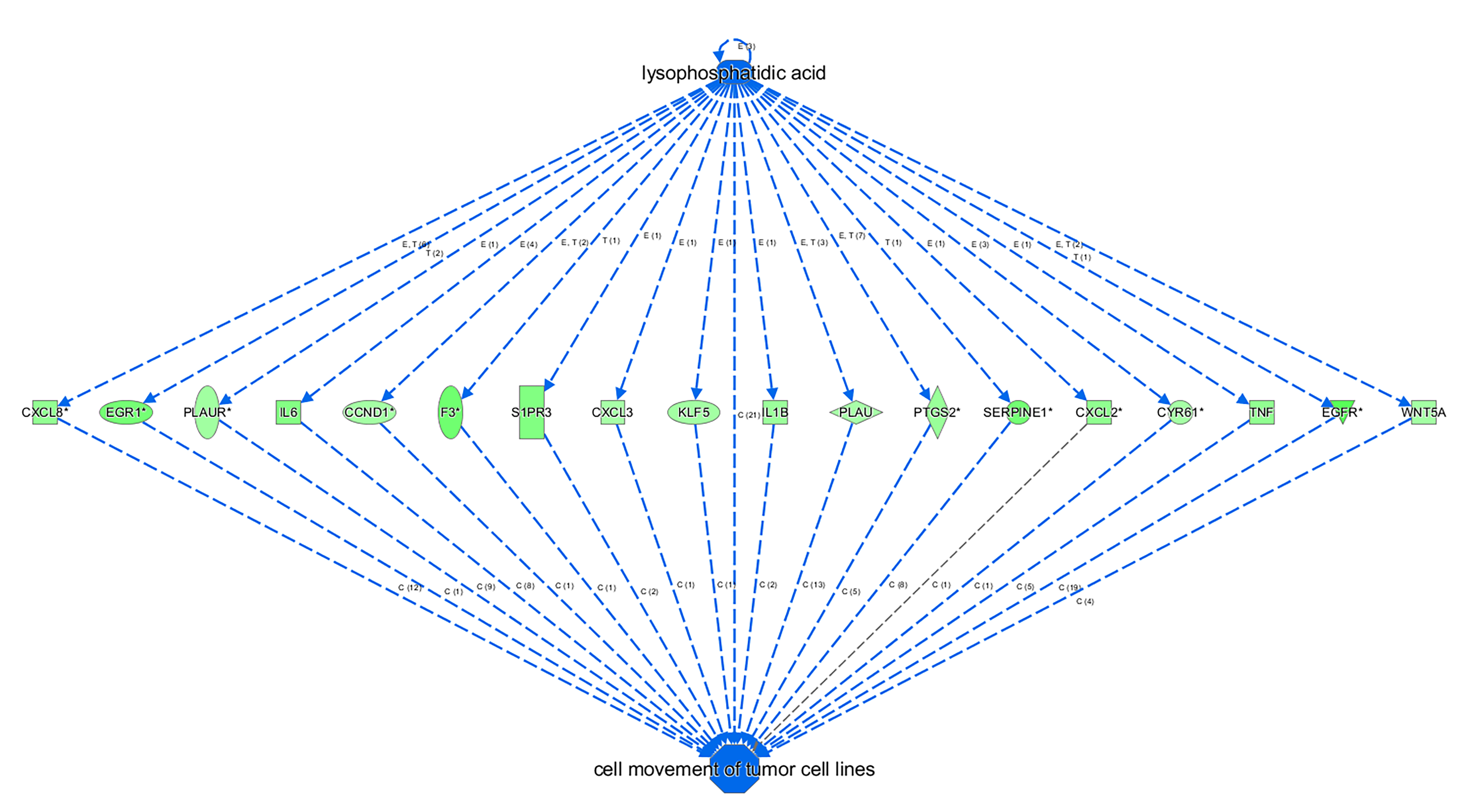

Supplement: Supplementary file 4 [file JCMM-24-1504-s004.png]
