# Supplementary material for: Importance of PNO1 for growth and survival of urinary bladder carcinoma: Role in core‐regulatory circuitry
Source: J Cell Mol Med. 2019 Dec 4;24(2):1504–15. doi: 10.1111/jcmm.14835 (PMC6991670; doi:10.1111/jcmm.14835)
Supplement: Supplementary file 5 [file JCMM-24-1504-s005.docx]

**SUPPLEMENTARY FIGURES**

**Supplementary Figure 1. Network map of the Acute Phase Response signaling pathway.** Purple color were highlighted molecules.

**Supplementary Figure 2. Network map of activated Acute Phase Response signaling pathway with activation/inhibition state of each gene supported by previous literatures.**

**Supplementary Figure 3. Interaction between the upstream regulatory factor (phorbol myristate acetate) and its directly related downstream molecules within the data set.** Orange/blue/yellow lines represented the consistent activation state/consistent inhibition state/inconsistent expression state of the downstream gene between literature and this microarray result as regulated by phorbol myristate acetate.

**Supplementary Figure 4. Regulatory effect network between lysophosphatidic acid and cell movement of tumor cell lines**.
